# Long Non-Coding RNA *lncWOX11a* Suppresses Adventitious Root Formation of Poplar by Regulating the Expression of *PeWOX11a*

**DOI:** 10.3390/ijms24065766

**Published:** 2023-03-17

**Authors:** Na Ran, Sian Liu, Haoran Qi, Jiali Wang, Tengfei Shen, Wenlin Xu, Meng Xu

**Affiliations:** 1 State Key Laboratory of Tree Genetics and Breeding, Co-Innovation Center for Sustainable Forestry in Southern China, Nanjing Forestry University, 159 Longpan Road, Nanjing 210037, China; njlydxrn@163.com (N.R.); HaoranQi@njfu.edu.cn (H.Q.); wangjiali971222@163.com (J.W.); tengfeishen@njfu.edu.cn (T.S.); wenlinxu.njfu@outlook.com (W.X.); 2 College of Horticulture and Plant Protection, Yangzhou University, Yangzhou 225000, China; sianliu@yzu.edu

**Keywords:** WUSCHEL-related homeobox protein, long non-coding RNA, CRISPR/Cas9, *cis*-regulatory module, *Populus*, protoplast

## Abstract

Long non-coding RNAs (lncRNAs), a class of poorly conserved transcripts without protein-encoding ability, are widely involved in plant organogenesis and stress responses by mediating the transmission and expression of genetic information at the transcriptional, posttranscriptional, and epigenetic levels. Here, we cloned and characterized a novel lncRNA molecule through sequence alignment, Sanger sequencing, transient expression in protoplasts, and genetic transformation in poplar. *lncWOX11a* is a 215 bp transcript located on poplar chromosome 13, ~50 kbp upstream of *PeWOX11a* on the reverse strand, and the lncRNA may fold into a series of complex stem–loop structures. Despite the small open reading frame (sORF) of 51 bp within *lncWOX11a*, bioinformatics analysis and protoplast transfection revealed that *lncWOX11a* has no protein-coding ability. The overexpression of *lncWOX11a* led to a decrease in the quantity of adventitious roots on the cuttings of transgenic poplars. Further, *cis*-regulatory module prediction and CRISPR/Cas9 knockout experiments with poplar protoplasts demonstrated that *lncWOX11a* acts as a negative regulator of adventitious rooting by downregulating the WUSCHEL-related homeobox gene *WOX11*, which is supposed to activate adventitious root development in plants. Collectively, our findings imply that *lncWOX11a* is essential for modulating the formation and development of adventitious roots.

## 1. Introduction

The formation and development of adventitious roots (ARs) is the key to the large-scale vegetative propagation of elite genotypes in many economically important woody species [1]. As ecologically and economically important trees in the Northern Hemisphere [2,3], the species of the genus *Populus* in Salicaceae mainly rely on cutting propagation to produce a large number of genotypically uniform plant materials [2] for not only timber and pulp production, but also the construction of bioenergy forests and shelter forests [4]. The adventitious rooting of tree cuttings, as a key criterion for selecting superior genotypes in the forestry industry, is a complex quantitative trait regulated by polygenes [5,6,7,8]. Despite enormous efforts over the last few decades to understand the anatomical, physiological, and biochemical mechanisms of adventitious rooting in forest trees, the genetic and molecular mechanisms of AR formation remain elusive.

Our current understanding of the molecular regulation mechanism of AR formation mainly comes from the progress of *Arabidopsis thaliana* [9], *Oryza sativa* [10], and *Zea mays* [11]. The basis of AR organogenesis is the totipotency and plasticity of cells, and the essence is the fate transitions of cells induced by trauma [12], environment [13], and physiological signals [14]. Plant-specific WUSCHEL-related homeobox (WOX) transcription factors [15], especially *WOX11* [16], play critical regulatory roles in this process. *Arabidopsis WUSCHEL* (*AtWUS*) is the prototypical member of the *WOX* gene family, while *AtWOX5* [17], as a close homologue of *AtWUS*, is a root stem cell organizer. Both genes were reported to be functionally exchangeable in regulating stem cell maintenance in both shoot and root contexts. *AtWOX11* and *AtWOX12* control the first-step cell fate transition during AR organogenesis by activating *LATERAL ORGAN BOUNDARIES DOMAIN 16* (*LBD 16*) and *WOX5/7* [18]. *AtWOX11* and auxin are involved in the transition of some vascular formative layers from xylem/woody stem cell production to the establishment of new lateral root primordia [19]. *OsWOX11*, the rice homologue of *AtWOX11*, has been reported to command the organogenesis of rice crown roots by participating in multiple regulatory pathways [20,21]. There are many conservative molecular regulatory pathways in the AR formation of trees and herbs; *WOX11*, *WOX5*, *LBD*, and other functional genes also play a central role in de novo AR primordia formation in trees. The overexpression of *PeWOX11a* and *PeWOX11b* not only increases the number of Ars, but also induces ectopic roots in transgenic poplar [22]. The *WOX11/12a-SMALL AUXIN-UP RNA 36* (*SAUR36*) module can mediate AR formation via auxin or salt stress pathways in poplar [23]. In *Populus*, woody taproots were treated with bending to induce the formation of new roots. During this process, *PnWOX11* was shown to be expressed during new lateral root formation at different developmental stages [24]. Overall, the rooting pathway mediated by *WOX11* may help many plant species create plastic root systems.

In recent years, increasing studies have focused on the involvement of long non-coding RNAs (lncRNAs) in plant organogenesis by mediating the transmission and expression of genetic information at the transcriptional, posttranscriptional, and epigenetic levels [25]. Unlike well-known microRNAs (miRNAs), lncRNAs are a class of poorly conserved transcripts with a length of more than 200 nucleotides and no protein-coding ability [26,27]. Most plant lncRNAs have specific secondary structures and spatiotemporal expression patterns, and their regulatory mechanisms are complex and diverse [28]. With the continuous updating of new sequencing technologies and biological prediction algorithms, the action trace of lncRNA has been found in many links between plant growth [29,30,31,32,33] and stress response [25,34]. The authenticity identification and annotation of these lncRNAs have been enormously challenging, and there is still a long way to go for further experimental verification and validation. To date, despite a large number of lncRNAs predicted in trees, knowledge about the biological functions and mechanisms of tree lncRNAs is still very limited.

According to the current study, lncRNAs play an important role mainly in the response of poplar to various abiotic stresses and in the development of woody tissue. An lncRNA–gene interaction network was constructed in poplar, deciphering that lncRNAs can respond to heat stress by regulating heat shock protein (HSP) family genes [35]. Another study showed that lncRNAs can regulate their target genes through RNA interference to increase photosynthetic recovery and protection while reducing DNA damage in plants from heat stress [36]. Exploring the physiological mechanisms of wood properties in poplars as they adapt to low-nitrogen environments provides a strategy for growing poplars in nitrogen-poor soils. Several studies have demonstrated the involvement of lncRNAs in this biological process, either directly or through the miRNAs–lncRNAs–mRNA pathway [37,38]. A number of lncRNAs have been identified in poplar that can participate in biological processes such as carbon metabolism, cellulose and lignin synthesis, and signal transduction by regulating their target genes [39,40]. Among them, lncRNA *PMAT* can control the tolerance and uptake of Pb2+ in root organs through the *PMAT–PtoMYB46–PtoMATE–PtoARF2* pathway [41]. The mechanism of the role of lncRNAs in poplar development still needs to be studied in depth.

In our previous study [22], two genes encoding *WOX11* transcription factors were identified and characterized through the rapid amplification of cDNA ends and genetic transformation in *Populus*. Overexpressing *PeWOX11a* or *PeWOX11b* in poplar would encourage more AR formation and stimulate ectopic root formation in aerial parts. Further research based on strand-specific RNA sequencing and preliminary validation experiments indicated that a large number of lncRNAs were involved in regulating the organogenesis of poplar ARs, and several potentially important lncRNAs may affect adventitious rooting by regulating the expression of *PeWOX11s* [42]. In this study, *lncWOX11a*, a 215 bp transcript located on poplar chromosome 13, ~50 kbp upstream of *PeWOX11a* on the reverse strand, was cloned and characterized, and detailed information about the stem–loop structures, expression patterns, coding ability, and biological functions of *lncWOX11a* was revealed. The overexpression of *lncWOX11a* led to a decrease in the quantity of ARs on the cuttings of transgenic poplars. Further *cis*-regulatory module prediction and CRISPR/Cas9 knockout experiments with poplar protoplasts demonstrated that *lncWOX11a* acts as a negative regulator of adventitious rooting by downregulating *PeWOX11a*.

## 2. Results

### 2.1. Isolation and Sequence Analysis of lncWOX11a

**Figure 1:**
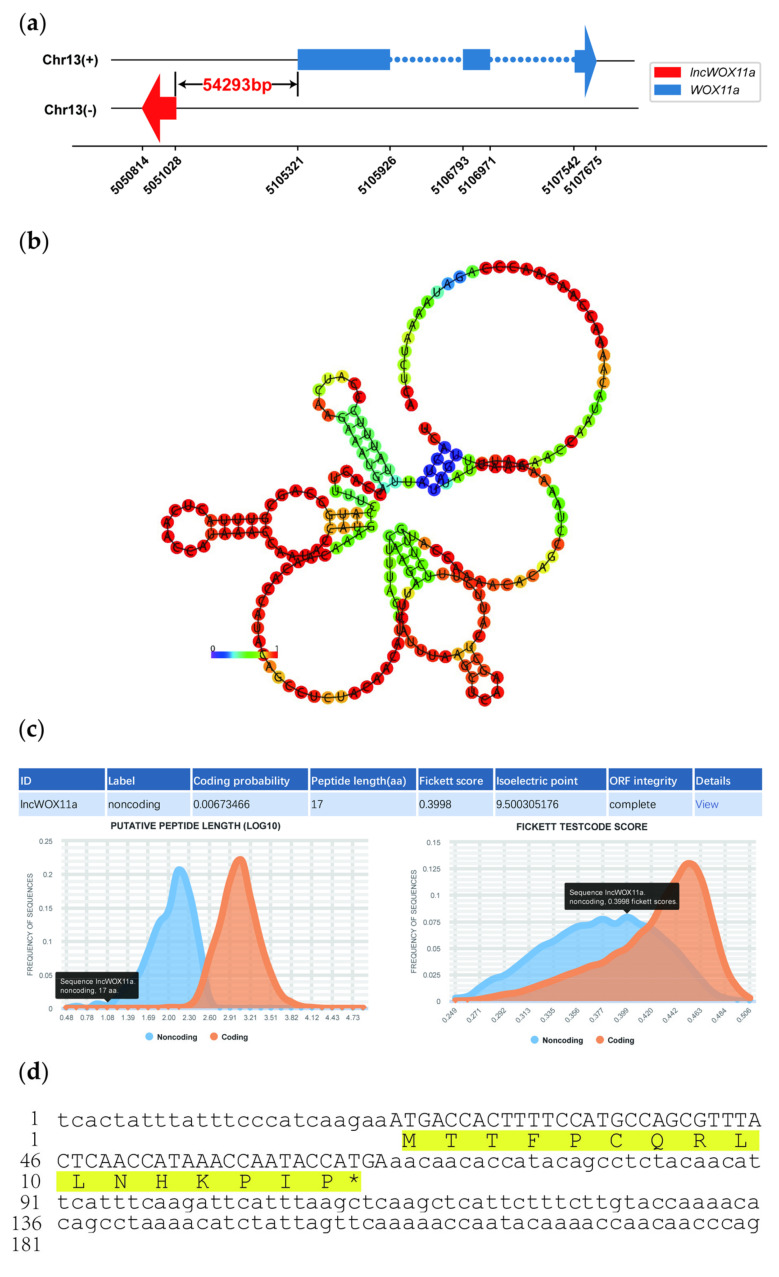
Sequence analysis of *lncWOX11a*. (**a**) Schematic representation of the *lncWOX11a–PeWOX11a* genomic region. (**b**) Prediction of the secondary structure of *lncWOX11a*. The different colors represent the level of minimum free energy. (**c**) Prediction of the coding ability of *lncWOX11a* by CPC2 software. (**d**) Nucleotide sequence of *lncWOX11a.*

Based on our previous strand-specific RNA sequencing data [42], DNA and cDNA sequences of the target lncRNA were cloned from *P. deltoides* × *P. euramericana* cv. ‘Nanlin 895’ roots, which were the same, with a length of 215 bp. *lncWOX11a* was located 54293 bp upstream of *PeWOX11a* (Figure 1a) on chromosome 13. The subcellular localization was predicted using lncLocator, and the results showed that *lncWOX11a* was localized in the nucleus. To investigate the conservation of the sequence, homology searches were performed on the NCBI, TAIR, and NONCODE databases. No similar sequences were retrieved, indicating that *lncWOX11a* was poorly conserved. The online RNAfold software was used to analyze the secondary structure of *lncWOX11a*, which had a complex secondary structure composed of multiple stem–loop structures. The minimum free energy (MFE) of *lncWOX11a* was predicted to be −11.40 kcal/mol, which indicates that it is relatively stable (Figure 1b). The prediction of miRNA binding sites for *lncWOX11a* using a plant miRNA target gene analysis tool revealed that *lncWOX11a* cannot bind to all the miRNAs in poplar, indicating that *lncWOX11a* cannot function by binding to miRNAs.

The coding ability is a crucial indicator for identifying lncRNAs. The results obtained with CPC2 software showed that *lncWOX11a* has no coding ability (Figure 1c). The ribosome is where mRNAs are translated into proteins, and the presence or absence of ribosome binding sites (RBSs) in an RNA sequence determines whether it can be translated successfully. The RBS was predicted using IRESite software, and no RBS was detected on *lncWOX11a* (Figure S1). As shown in Figure 1d, the small open reading frame (sORF) of *lncWOX11a* was only 51 bp in length and may encode 16 amino acids. A search of the BLASTP database for the 16 amino acids revealed no matching homologous sequences; in addition, a search of the Pfam database for the amino acids did not reveal any functional structural domains, suggesting, from a bioinformatics perspective, that the small peptide of *lncWOX11a* may not have the capacity to encode the protein.

### 2.2. Verification of the Coding Ability of lncWOX11a

**Figure 2:**
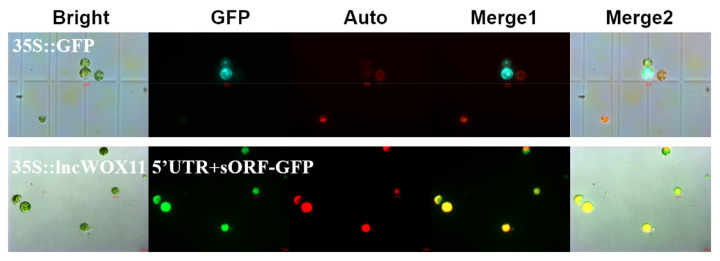
Verification of the coding ability of *lncWOX11a*. Protoplasts transiently expressing *lncWOX11a* were observed under a fluorescence microscope. Bright-field, green fluorescent protein (GFP), chlorophyll autofluorescence (Auto), merged 1, and merged 2 images are shown. Scale bar = 10 µm.

Recent studies have reported that some lncRNAs predicted by bioinformatics software have false positives, and these were later experimentally verified to have the ability to encode peptides and then act as peptides. Therefore, we further verified the coding ability of *lncWOX11a* by transient expression in protoplasts. We constructed a *35S::lncWOX11a* 5’UTR+sORF-GFP fusion vector using Gateway technology and transferred it into poplar protoplasts. The results showed fluorescence of the control *35S::GFP* construct in the nucleus, cell membrane, and cytoplasm of poplar protoplasts, whereas after *35S::lncWOX11a* 5’UTR+sORF-GFP transfection, only chloroplast autofluorescence and no green fluorescent protein (GFP) fluorescence were detected (Figure 2). All results were replicated in three independent experiments, proving that *lncWOX11a* is a long non-coding RNA.

### 2.3. Organ-Specific Expression of lncWOX11a

**Figure 3:**
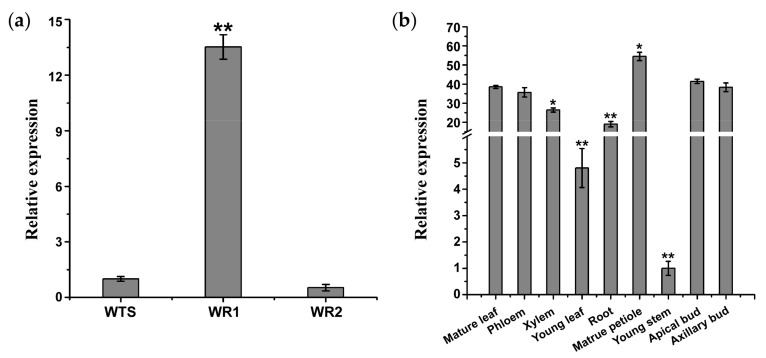
Expression patterns of *lncWOX11a* in different organs of poplar. (**a**) *lncWOX11a* was specifically expressed in 1-week-old roots. WTS represents the stems of 30-day-old tissue-cultured seedlings. WR1 and WR2 represent 1-week-old roots and 2-week-old roots of tissue-cultured seedlings, respectively. (**b**) *lncWOX11a* was mainly expressed in mature organs. Mature leaves were picked in July of the study year, and all other organs were picked at the end of March of the test year, One-way ANOVA was used to identify statistically significant differences. The data are presented as the means ± SEs. The “*” above the histogram indicates significance, “*” represents *p* < 0.05, and “**” represents *p* < 0.01.

The abundance of *lncWOX11a* transcripts in various poplar organs was assessed by quantitative real-time polymerase chain reaction (qRT–PCR). The results showed that *lncWOX11a* was mainly expressed in 1-week-old roots from cuttings and showed weak expression in 2-week-old roots and stems (Figure 3a). In addition, the expression patterns of *lncWOX11a* were ubiquitous in nine organs, and no significant organ-specific expression pattern was detected in the roots. Despite being more highly expressed in 1-week-old roots, *lncWOX11a* was very highly expressed in other organs. The lowest *lncWOX11a* expression was found in young stems, followed by young leaves. The expression of *lncWOX11a* was significantly higher in the other seven organs and was highest in the mature petiole (Figure 3b).

### 2.4. The Overexpression of lncWOX11a Inhibits AR Development

**Figure 4:**
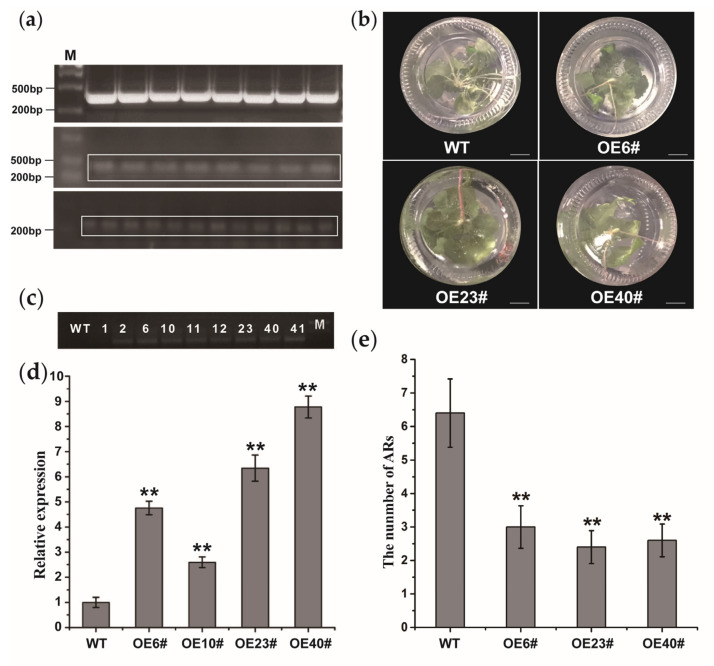
Molecular identification of transgenic poplar overexpressing *lncWOX11a.* (**a**) Construction of the *lncWOX11a* overexpression vector. The graph shows the BP reaction, LR reaction, and *Agrobacterium* tumefaciens detection. (**b**) The number of ARs in transgenic poplars at 30 days was lower than in WT plants at the same age. Scale bar = 10 mm. (**c**) DNA amplification was used to identify nine transgenic plants. (**d**) Detection of *lncWOX11a* expression in WT and OE transgenic lines. (**e**) Statistics on the number of ARs in the OE transgenic lines. One-way ANOVA was used to identify statistically significant differences. The data are presented as the means ± SEs. The “*” above the histogram indicates significance, and “**” represents *p* < 0.01.

Previous studies have shown that *WOX11a* has a function in AR formation [22]. To further confirm the potential role of *lncWOX11a* in woody plants, we transformed the *35S::lncWOX11a* construct into the hybrid poplar ‘Shanxin’. The overexpression vector *35S::lncWOX11a* was constructed using the gateway method, as shown in Figure 4a. The *lncWOX11a* sequence was ligated to the entry vector by a BP reaction and was then transferred into the overexpression vector by an LR reaction. Afterwards, the correctly sequenced *35S::lncWOX11a* vector was transferred into *Agrobacterium* EHA105. Nine lines of transgenic plants showing healthy growth were chosen for DNA detection. The results of agarose gel electrophoresis showed that the wild type (WT) and OE1# lines did not show any amplified bands after PCR amplification, whereas bands were detected with the other eight lines (Figure 4c). We randomly selected four OE transgenic lines for further detection. According to the qRT–PCR results, we found that the expression level of *lncWOX11a* in the four OE transgenic lines was 2–8 times higher than that in the WT, which tentatively implies that the *35S::lncWOX11a* construct was integrated into the poplar genome (Figure 4d).

To determine the effect of *lncWOX11a* overexpression on the growth of transgenic lines, we selected four independent lines (OE6#, OE23#, and OE40# lines) for subsequent phenotypic observation experiments. For each strain, we set up five biological replicas separately. One-month-old transgenic and WT lines were continuously cultured in MS medium. Then, we observed the growth of ARs in the transgenic lines and counted the number of roots produced. The root architecture of the OE lines was significantly different from that of the WT plants, and a 1-fold reduction in the average number of ARs (including taproots and lateral roots) was detected in the OE lines (Figure 4b,e). Specifically, 10 days after regeneration, the WT line began to form ARs near the stem, and no growth dominance was observed in the transgenic lines. After 30 days, the number of ARs continued to increase in the WT and OE transgenic lines. However, the OE transgenic lines had certain advantages in terms of plant height. These results indicated that *lncWOX11a* might negatively modulate AR formation.

### 2.5. The cis-Regulation Module for lncWOX11a

**Figure 5:**
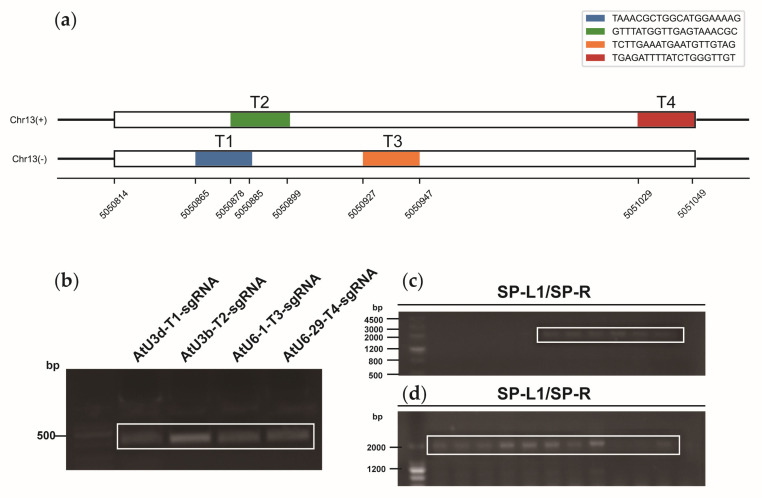
Construction of the CRISPR/Cas9 vectors for *lncWOX11a*. (**a**) Target sequences of CRISPR/Cas9 in *lncWOX11a*. The white region represents the exon of *lncWOX11a*. Two separate targets were designed for the positive and negative sense strands, and each had a length of 20 bp. (**b**) Construction of the sgRNA intermediate vector. The second round of PCR products was detected by agarose gel electrophoresis. (**c**) Construction of the pYLCRISPR/Cas9pUbi-N vector. (**d**) Construction of the pYLCRISPR/Cas9p35S-N vector. Positive clones were detected by colony PCR using primers from the vector with the universal primers SP-R and SP-L1, and the white box indicates the fragment containing four tandem sgRNA expression cassettes.

**Figure 6:**
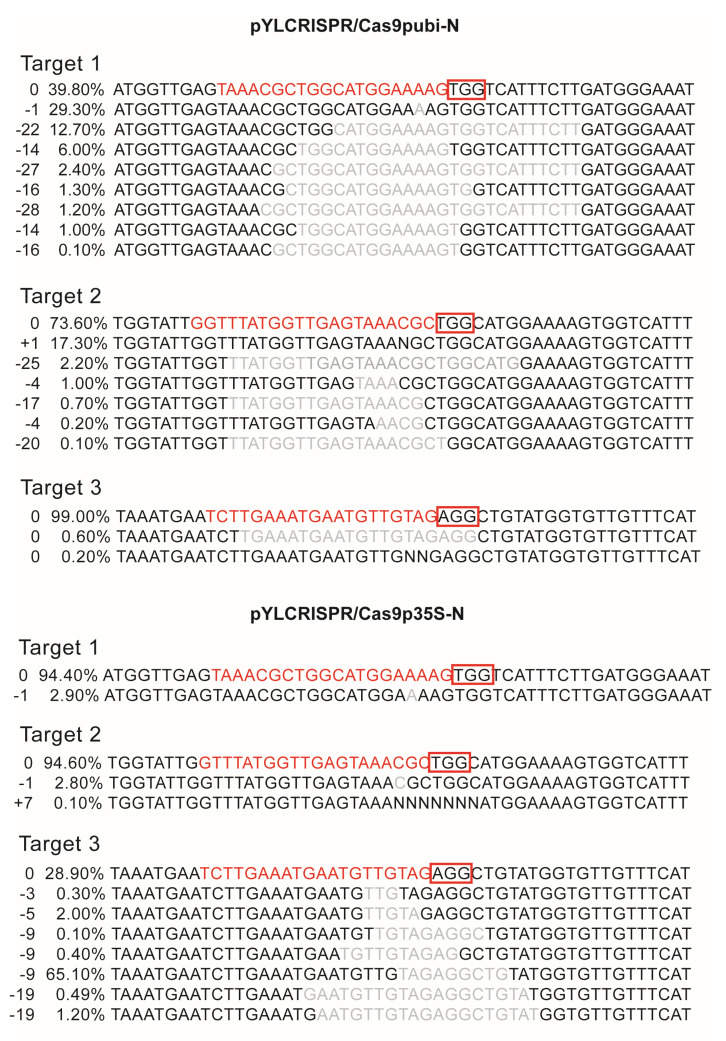
Evaluation of the knockout efficiency of the pYLCRISPR/Cas9pubi-N and pYLCRISPR/Cas9p35S-N vectors for *lncWOX11a*. The first column of data represents missing or added cases, and the second column of data shows the knockout efficiency. The red font represents target sequences, and the grey font indicates deletions. Protospacer-adjacent motifs (PAMs; NGGs) are shown in red boxes.

Based on the four target sites (Figure 5a), we constructed knockout vectors. The four target sequences were constructed into four sgRNA expression cassettes using the overlapping PCR method (Figure 5b); the four expression cassettes were then cloned into knockout vectors, and colony PCR was performed using the universal primers SP-L1 and SP-R to detect positive clones (Figure 5c,d). Genomic DNA from protoplasts was sequenced, and the sequencing data were subsequently analyzed using Synthego software to identify base insertions or deletions near the target sites. The knockout efficiency of *lncWOX11a* in protoplasts using the two vectors did not differ considerably. At targets 1 and 2, the knockout efficiency of the pYLCRISPR/Cas9pUbi-N vector was more noticeable, and at target 3, the knockout efficiency of the pYLCRISPR/Cas9p35S-N vector was higher. However, no editing events were discovered close to the sequence of target T4 (Figure 6).

**Figure 7:**
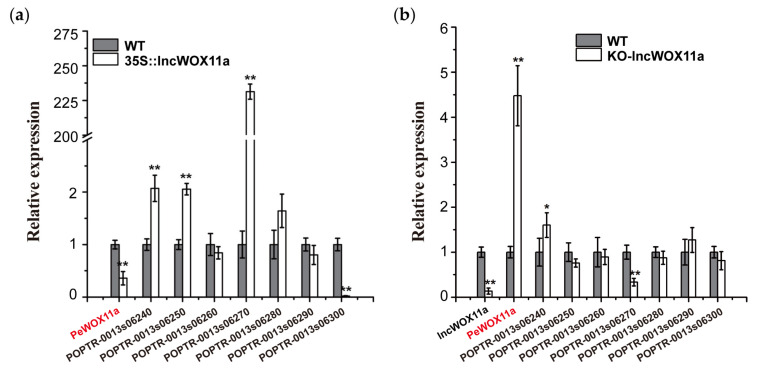
Expression levels of *cis*-regulated target genes of *lncWOX11a*. (**a**) Expression levels of *cis*-regulated target genes of *lncWOX11a* in *35S::lncWOX11a* poplar. (**b**) Expression level of the *cis*-regulated target gene of *lncWOX11a* in poplar protoplasts after the knockout of *lncWOX11a*. One-way ANOVA was used to identify statistically significant differences. The data are presented as the means ± SEs. The “*” above the histogram indicates significance, “*” indicates *p* < 0.05, and “**” indicates *p* < 0.01.

Based on a genome annotation analysis within 100 kb upstream or downstream of *lncWOX11a*, we detected eight target genes *cis*-regulated by *lncWOX11a*. These putative targets included the *PeWOX11a* gene, which has been shown to be important for AR formation [22]. We used qRT–PCR to monitor the expression of these eight genes in the OE40# line to detect the regulation between *lncWOX11a* and the target genes. As shown in Figure 7a, the expression of these target genes varied significantly in the OE40# line. Among them, the expression of three target genes increased significantly after the overexpression of *lncWOX11a*; specifically, *POPTR_0013s06270* showed the highest expression, and the expression of *PeWOX11a* and *POPTR-0013s06300* was markedly lower than that in the WT. The results illustrated a suppressive effect of the overexpression of *lncWOX11a* on the expression of *POPTR_0013s06300* and *PeWOX11a*.

The expression levels of *lncWOX11a* and the target genes were examined in protoplasts that had been transduced into knockout vectors by qRT–PCR. First, the knockout experiment was successful, as evidenced by the decreased expression of *lncWOX11a*. Among the eight target genes, *PeWOX11a* showed a large increase in expression, whereas the target gene *POPTR_0013s06240* showed a small increase (Figure 7b). *POPTR_0013s06270* exhibited a decreasing trend relative to the expression of WT, whereas the others did not change significantly and may not be regulated by *lncWOX11a*. Based on these results, combined with the target gene expression quantity after overexpression, we found that *lncWOX11a* showed the same trend as *POPTR_0013s06270*, implying a positive regulatory relationship between them. *PeWOX11a* exhibited the opposite pattern, demonstrating that they were negatively regulated by one another. In summary, we preliminarily suppose that *lncWOX11a* may inhibit AR development in poplar by downregulating *PeWOX11a*.

## 3. Discussion

Exploring the molecular mechanisms of AR development provides ideas for large-scale asexual reproduction strategies for some woody species. A growing body of research suggests that lncRNAs are essential regulators of a wide range of biological processes. To understand how lncRNAs play a role in AR formation and development, we investigated the biological function of *lncWOX11a* in hybrid poplars and the regulatory relationship between *lncWOX11a* and *WOX11a*.

In this study, a 215 bp transcript located on poplar chromosome 13 was cloned from ‘Nanlin 895’, and a sequence analysis of *lncWOX11a* was performed using online bioinformatics software. The ability to code is a critical requirement for determining whether a long transcript is an lncRNA. The absence of RBS on *lncWOX11a* was detected by bioinformatics software, indicating that it could not be translated into a protein. lncRNAs have several major mechanisms of action, such as guide molecules [43] and scaffold molecules [44]. In addition, lncRNAs can act as decoy molecules that can bind to miRNAs [44]. miRNAs can target and repress the expression of protein-coding genes. When such lncRNAs bind to miRNAs, they are able to compete with the target genes of miRNAs, which reduces the quantity of miRNA-targeting genes and alleviates the repression of protein-coding genes by miRNAs. These lncRNAs are also regarded as competing endogenous RNAs (ceRNAs) [45]. The prediction of miRNA binding sites revealed that *lncWOX11a* does not function by acting as a decoy molecule. Furthermore, by conducting transient expression experiments in protoplasts, we concluded that *lncWOX11a* is a novel non-coding RNA.

The important role of the *WOX11* gene in root organ development is evident from a large number of reports [16]. *PagWOX11/12a* can modulate ROS scavenging by regulating the expression of *PagCYP736A12* to enhance salt tolerance in poplar [46], while ROS has been shown to be closely associated with the development of ARs in several species [47,48]. Overall, the way in which *WOX11* promotes adventitious rooting in angiosperms is at least evolutionarily conserved, and it could be a vital molecular tool to promote rooting from stem or leaf cuttings. Therefore, functional studies and mechanistic analyses of the *WOX11* gene in poplar still need to be continued. In plants and other eukaryotes, the expression level of lncRNA is usually much lower than that of mRNA [49]. We selected various poplar organs to measure the expression pattern of *lncWOX11a*. The expression of *lncWOX11a* was generally high in various organs, and no significant organ-specific expression pattern was found in the roots. In addition, the overexpression of *lncWOX11a* in poplar suppressed AR formation to some extent, which complemented the biological function of *WOX11a*.

Regulating the expression of downstream functional genes is a valuable mechanism through which lncRNAs participate in plant growth. lncRNAs can regulate the expression of nearby genes in *cis* and distant genes in *trans* [50,51]. By prediction, we found that *lncWOX11a* may regulate some protein-coding genes by *cis*-regulating action. Although the CRISPR/Cas9 editing system is gradually becoming a critical instrument for genetic breeding and gene function research, a stable genetic transformation system is still lacking for many forest trees. Therefore, we performed CRISPR/Cas9 knockout experiments using poplar protoplasts. Accordingly, we elaborated on the relationship between *lncWOX11a* and the potential protein-coding genes (Figure S2). Among these putative target genes, the gene annotations showed that *POPTR_0013s06300* encodes a Class III peroxidase PRX70 protein; *POPTR-0013s06240* belongs to glycosyltransferase family 14 (GT14). Glycosyltransferase (GT) is a key enzyme in the synthesis of plant cell wall polysaccharides and glycoproteins. To date, studies on the GT14 family have focused on poplar, *A. thaliana*, *O. sativa* [52,53], and others. Combined with the above-described experimental results, we speculate that *lncWOX11a* may have a regulatory relationship with *PeWOX11a* and *POPTR_0013s06270*. In addition, we found that the expression of the *POPTR-0013s06240* gene increased after both *lncWOX11a* overexpression and knockdown. Based on this result, we speculate that a direct regulatory relationship may not exist between *lncWOX11a* and *POPTR-0013s06240*. In our previous study, the *35S::lncWOX11* plasmid was transiently expressed in protoplasts, after which the expression of *PeWOX11a* was found to be upregulated [42]. This experiment builds on previous studies to further validate the biological function of *lncWOX11a* in poplar through experiments such as genetic transformation and CRISPR/Cas9 gene knockdown. Finally, we proposed a hypothesis that *lncWOX11a* may exert a suppressive effect on *PeWOX11a*, thus inhibiting the growth and development of ARs in transgenic plants. However, the genetic evidence and the regulatory module between *lncWOX11a* and *PeWOX11a* must be better confirmed in subsequent experiments.

## 4. Materials and Methods

### 4.1. Plant Materials and Growth Conditions

Tissue-cultured seedlings of a cultivated variety (*Populus deltoides × P. euramericana* cv. ‘Nanlin 895’) were used as the materials. The seedlings were grown on Murashige and Skoog (MS) medium (pH 5.8) in a humid chamber at a temperature of 25/18 °C (day/night). The photoperiod was 16/8 h per day (light/dark), and the relative humidity was 60–80%. Tissue-cultured ‘Nanlin 895’ seedlings were used for gene cloning, and the leaves from 40-day-old seedlings were used for transient protoplast expression. Nine organs from 2-year-old ‘Nanlin 895’ poplars were used for RNA extraction to measure the transcript abundance of *lncWOX11a*. The samples were stored in a freezing chamber at −80 °C for further experiments. We used hybrid poplar (*P. davidiana × P. bolleana* cv. ‘Shanxin’) as the transformation acceptor plantlets in this study and cultivated it as described above.

### 4.2. Cloning and Sequence Analysis

We designed amplification-specific primers (Table S1) for cloning based on the *lncWOX11a* sequences obtained in our previous study [42]. First, RNA and DNA were extracted separately from 1-week-old roots of ‘Nanlin 895’ tissue-cultured seedlings. Genomic DNA was obtained using a DNeasy Plant Mini Kit (Tiangen, China). Total RNA was separated using the RNAprep Pure Plant Plus kit (Tiangen, China), and cDNA was then synthesized from RNA using the PrimeScript RT Master Mix Kit (Takara, China). To explore whether lncRNAs homologous to *lncWOX11a* exist in other species, cDNA sequence similarity comparisons were performed with NCBI BLASTN (https://blast.ncbi.nlm.nih.gov/ (accessed on 10 March 2022)), the *Arabidopsis* TAIR database (https://www.arabidopsis.org/ (accessed on 10 March 2022)), and the NONCODE database (http://www.noncode.org/ (accessed on 10 March 2022)) with the threshold set to E < 0.005; the subcellular localization was predicted using lncLocator; the miRNA binding sites were predicted using psRNATarget (https://www.zhaolab.org/ (accessed on 15 July 2022)); RNAfold (http://rna.tbi.univie.ac.at/cgi-bin/RNAWebSuite/RNAfold.cgi (accessed on 15 July 2022)) was used to predict the secondary structure; CPC2 software (http://cpc2.cbi.pku.edu.cn/ (accessed on 16 July 2022)) was used for the assessment of the coding ability; the ORF was searched using the NCBI ORF finder (https://www.ncbi.nlm.nih.gov/ (accessed on 16 July 2022)); and IRESite software (http://www.iresite.org/ (accessed on 20 August 2022)) was aimed at determining RBSs.

### 4.3. Transient Expression in Poplar Protoplasts

Some studies have shown that carrying the 5’UTR of a gene at the 5’ end of the ORF can facilitate the translation of the gene. To experimentally validate the coding ability of *lncWOX11a*, we designed primers (Table S1) targeting *lncWOX11a* 5’UTR+sORF-GFP. PCR amplification was first performed using DNA polymerase, and the sequence was obtained by sequence verification. The correct sequence was transferred to the p2GWF7.0 vector carrying GFP using Gateway technology (Invitrogen) following the manufacturer’s protocol. We used a method that optimized the type and concentration of enzymes and the digestion time for the extraction of poplar protoplasts [54]. Finally, the plasmids were introduced into poplar protoplasts using the polyethylene glycol (PEG)-mediated transient expression system [54]. The fluorescence of the GFP tags was monitored under a fluorescence microscope (Scope A1 Carl Zesiss, Jena, Germany).

### 4.4. Quantitative Real-Time Quantification

Quantitative real-time polymerase chain reaction (qRT–PCR) aimed to assess the expression pattern of *lncWOX11a* in diverse organs from ‘Nanlin 895’. Nine organs, including the xylem, phloem, petiole, leaf, root, stem, and bud organs, were taken from 2-year-old ‘Nanlin 895’ poplar. First, total RNA was isolated from nine different organs, and cDNA was then synthesized from RNA. qRT–PCR was performed with an ABI ViiA™ 7 real-time PCR system using the PowerUp™ SYBR™ Green Master Mix (Applied Biosystems, Carlsbad, CA, USA) following the manufacturer’s manual. The 2^−ΔΔCT^ value between the target gene and the reference, *18S ribosomal RNA* (*18S*) [55], was used to compute the relative expression [56]. One-way ANOVA was used to examine statistically significant differences. The primers for qRT–PCR were designed using oligo6 and are provided in Supplementary Table S1.

### 4.5. Generation of Transgenic Poplar Lines

The *lncWOX11a* sequence was cloned into pH35GS using the Gateway System to construct the overexpression vector. The leaves from 40-day-old tissue-cultured ‘shanxin’ seedlings were used as the transformation acceptor plantlets. The *35S::lncWOX11a* plasmid was introduced into EHA105 and used for the genetic transformation of hybrid poplar ‘Shanxin’ using the *Agrobacterium*-based leaf disc method [57,58]. The OE lines were obtained after screening in MS medium with kanamycin. Afterward, amplification reactions at the DNA and RNA levels were performed on all putative OE lines and WT controls. The DNA of the transgenic plants was extracted for PCR amplification using p35SF on the promoter as the forward primer and the downstream primer of *lncWOX11a* as the reverse primer.

### 4.6. Target Gene Prediction

Based on genome annotation, genes located within 100 kb upstream or downstream of *lncWOX11a* were chosen as candidate target genes in the *cis*-regulatory mechanism [42]. The *cis*-regulatory module of *lncWOX11a* was mapped using Cytoscape software.

### 4.7. Construction of the pYLCRISPR/Cas9-lncWOX11a Vector

The CRISPR/Cas9 system [59] was used to confirm the regulatory module of *lncWOX11a*. We designed four sgRNA sequences that targeted *lncWOX11a* using the online website CRISPR-GE [59] (http://skl.scau.edu.cn/ (accessed on 20 March 2022)). The four target sites were ligated to the four sgRNA intermediate vectors by overlapping PCR, and the sgRNAs of the four target sites were actuated by the AtU3b, AtU3d, AtU6-1, and AtU6-29 promoters from *A. thaliana*. The four correct sgRNA expression cassettes were cloned into the pYLCRISPR/Cas9pUbi-N and pYLCRISPR/Cas9p35S-N vectors to complete the constructs by Golden Gate ligation [60,61]. The control and knockout vectors were transferred into poplar protoplasts, and the genomic DNA was then isolated from the plasmid-transferred protoplasts. The primers that contained the four target sites were used to perform PCR amplification and were then sequenced by the Sanger method. The data were analyzed using Synthego software to identify base insertions or deletions near the target sites, and additional experiments were subsequently conducted. All the primers needed to construct the knockout vectors are shown in Supplementary Table S2.

## 5. Conclusions

In conclusion, we isolated and cloned *lncWOX11a* from ‘Nanlin 895’, which is 215 bp in length. Both bioinformatic analysis and transient expression confirmed that *lncWOX11a* has no ability to encode proteins. Through genetic transformation in poplar, we elucidated the role of *lncWOX11a* in the regulation of AR development. The regulatory relationship between *lncWOX11a* and its target genes was explained by CRISPR/Cas9 knockout experiments in poplar protoplasts. Finally, we hypothesized that *lncWOX11a* could repress the expression of *PeWOX11a* to negatively regulate AR development.

## Data Availability

All the data are shown in the main manuscript and in the Supplementary materials.

## Supplementary Materials

The following supporting information can be downloaded at: https://www.mdpi.com/article/10.3390/ijms24065766/s1.
