# Supplementary material for: Long Non-Coding RNA lncWOX11a Suppresses Adventitious Root Formation of Poplar by Regulating the Expression of PeWOX11a
Source: Int J Mol Sci. 2023 Mar 17;24(6):5766. doi: 10.3390/ijms24065766 (PMC10057709; doi:10.3390/ijms24065766)
Supplement: Supplementary file 1 [file ijms-24-05766-s001.zip › Figure S1.pdf]

**Sequences producing significant alignments**	**bits**	**E Value**
IRESite_Id:489 plasmid pR-deltaEMCV-L1_ORF2_-201-1-F with functi.	31	0.019
IRESite_Id:496 plasmid pR-deltaEMCV-L1_ORF1_-400-1-F with functi.	31	0.019
IRESite_Id:490 plasmid pR-deltaEMCV-L1_ORF1_-400-1-F with functi.	31	0.019
IRESite_Id:494 plasmid pR-deltaEMCV-L1_ORF1_-101-1-F with functi.	31	0.019
IRESite_Id:244 plasmid pbetaGAL/XIAP/CAT with functional XIAP IR..	26	0.71
IRESite_Id:632 plasmid pbetaGAL/rc_XIAP/CAT with defective rc_XI...	26	0.71
IRESite_Id:491 AQP4 IRES	23	8.0
IRESite_Id:541 plasmid pBiCAQP4 with functional AQP4 IRES from A.	23	8.0
IRESite_Id:488 plasmid pBiCAQP4 with functional AQP4 IRES from A.	23	8.0
IRESite_Id:475 plasmid (deltaCMV)BiCAQP4 with functional AQP4 IR.	23	8.0
IRESite_Id:437 RhPV_5NCR IRES	21	27

Figure S1. Prediction of the RBS for *lncWOX11a* by IRESite software
