# Supplementary material for: Long Non-Coding RNA lncWOX11a Suppresses Adventitious Root Formation of Poplar by Regulating the Expression of PeWOX11a
Source: Int J Mol Sci. 2023 Mar 17;24(6):5766. doi: 10.3390/ijms24065766 (PMC10057709; doi:10.3390/ijms24065766)
Supplement: Supplementary file 1 [file ijms-24-05766-s001.zip › Table S1.pdf]

Table S1. Primers used for amplification of *lncWOX11a*

Primer ID	Primer Sequences（5'–3'）
lncWOX11a-F	TCACTATTTATTTCCCATCAAGAAA
lncWOX11a-R	TGAGATTTTATCTGGGTTGTTGGT
lncWOX11a 5'UTR+sORF-F	TCACTATTTATTTCCCATCA
lncWOX11a 5'UTR+sORF-R	TGGTATTGGTTTATGGTTGA
lncWOX11a qRT-PCR F	TCACTATTTATTTCCCATCAAGAAA
lncWOX11a qRT-PCR R	TGAGATTTTATCTGGGTTGTTGGT
PeWOX11a qRT-PCR F	GCCACTTGACATAAAAGCAA
PeWOX11a qRT-PCR R	TTCACCATGATGCAAGCTCT
POPTR_0013s06240 qRT-PCR F	GGCCTGGTGGGAAATGTTTGGA
POPTR_0013s06240 qRT-PCR R	CCCATTTGCAGGTCCTCAGCA
POPTR_0013s06250 qRT-PCR F	GTGTGCCAGGAGTGGCAAGA
POPTR_0013s06250 qRT-PCR R	ACTCTCATCTGCATTGCCGCTT
POPTR_0013s06260 qRT-PCR F	TGGAGGGATCCGAGGGCTAC
POPTR_0013s06260 qRT-PCR R	TGTCGTCACGGAACCTTGGG
POPTR_0013s06270 qRT-PCR F	CTTTGGGTCCAGCCCAGCAA
POPTR_0013s06270 qRT-PCR R	GCCTTGCTCCTCCTCATGCT
POPTR_0013s06280 qRT-PCR F	TGCCTCAGGATGCTGTGATTTCG
POPTR_0013s06280 qRT-PCR F	GCTCCAGTTCCCGGGTAGTCT
POPTR_0013s06290 qRT-PCR F	TTTCCGGCCGAACCACTAGC
POPTR_0013s06290 qRT-PCR R	CACTCGCATTGCAGCTTGGC
POPTR_0013s06300 qRT-PCR F	ACAGTTGCAACCAGAGATGCTGT
POPTR_0013s06300 qRT-PCR R	GTCCACTTGTGGTCCAGGCA
18s-F	TCAACTTTCGATGGTAGGATAGTG
18s-R	CCGTGTCAGGATTGGGTAATTT
