# Supplementary material for: Long Non-Coding RNA lncWOX11a Suppresses Adventitious Root Formation of Poplar by Regulating the Expression of PeWOX11a
Source: Int J Mol Sci. 2023 Mar 17;24(6):5766. doi: 10.3390/ijms24065766 (PMC10057709; doi:10.3390/ijms24065766)
Supplement: Supplementary file 1 [file ijms-24-05766-s001.zip › Table S2.pdf]

Table S2. Primers employed for *lncWOX11a* knockout vector construction and detection

Title 1	Primer Sequences（5'–3'）
U-F	CTCCGTTTTACCTGTGGAATCG
gR-R	CGGAGGAAAATTCCATCCAC
Pps-R	TTCAGAGGTCTCTACCGACTAGTCACGCGTATGGAATCGGCAGCAAA
Pgs-2	AGCGTGGGTCTCGTCAGGGTCCATCCACTCCAAGCTC
Pps-2	TTCAGAGGTCTCTCTGACACTGGAATCGGCAGCAAAGG
Pgs-3	AGCGTGGGTCTCGTCTTCACTCCATCCACTCCAAGCTC
Pps-3	TTCAGAGGTCTCTAAGACTTTGGAATCGGCAGCAAAGG
Pgs-4	AGCGTGGGTCTCGAGTCCTTTCCATCCACTCCAAGCTC
Pps-4	TTCAGAGGTCTCTGACTACATGGAATCGGCAGCAAAGG
SP-L1	GCGGTGTCATCTATGTTACTAG
SP-L2	GTCGTGCTCCACATGTTGACC
SP-R	TGCAATAACTTCGTATAGGCT
Cas9-F	CTGACGCTAACCTCGACAAG
Cas9-R	CCGATCTAGTAACATAGATGACACC
Testing-F	TCACTATTTATTTCCCATCAAGAAA
Testing-R	TGAGATTTTATCTGGGTTGTTGGT
