# Supplementary figures and images for: Long Non-Coding RNA lncWOX11a Suppresses Adventitious Root Formation of Poplar by Regulating the Expression of PeWOX11a
Source: Int J Mol Sci. 2023 Mar 17;24(6):5766. doi: 10.3390/ijms24065766 (PMC10057709; doi:10.3390/ijms24065766)

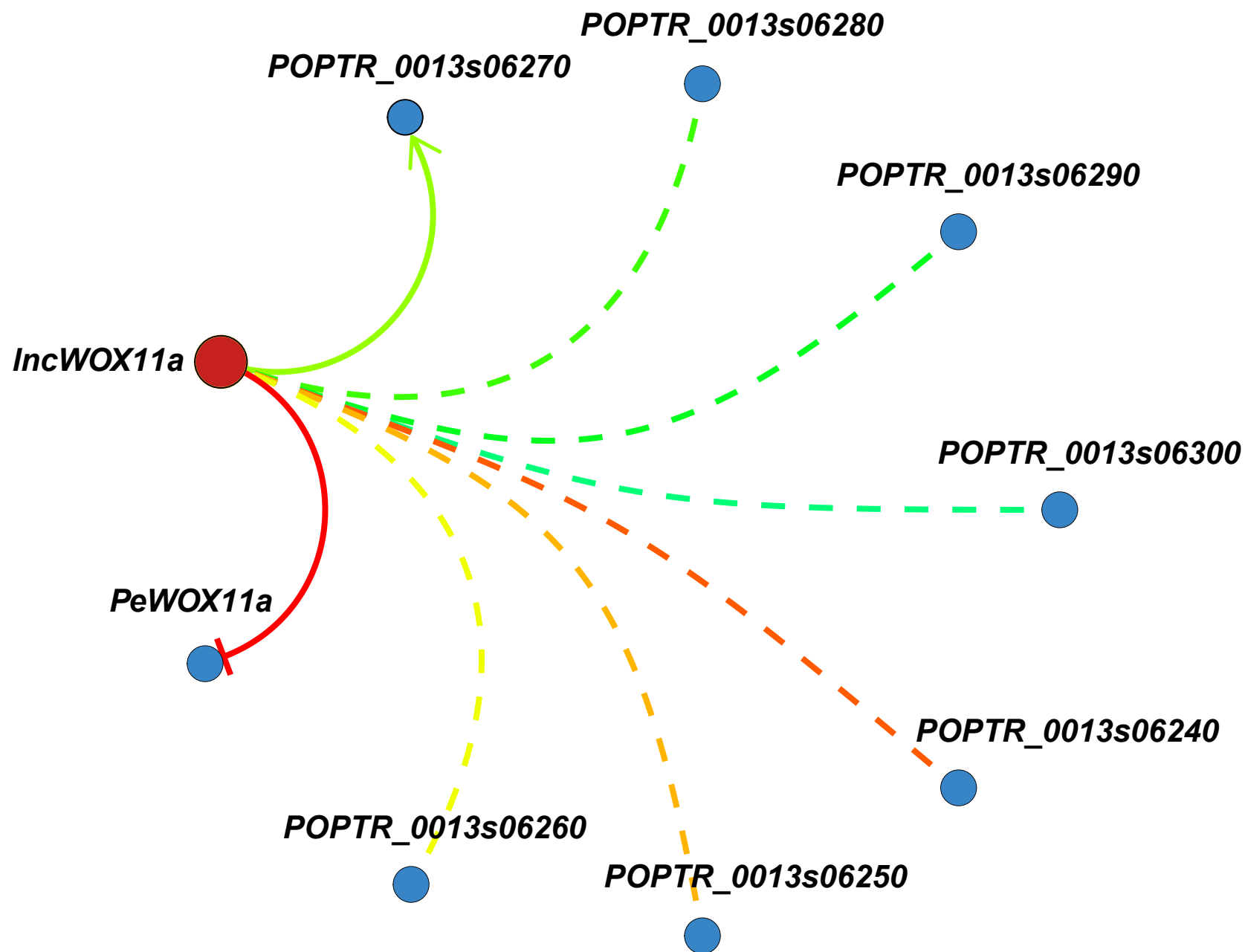

**Figure S2.** *Cis*-regulated network of *lncWOX11a*.

Supplement: Supplementary file 1 [file ijms-24-05766-s001.zip › figure S2.pdf]
